# Liver tissues oxidative status, epigenetic and molecular characteristics in rats administered magnetic and microwave treated water

**DOI:** 10.1038/s41598-023-31168-9

**Published:** 2023-03-16

**Authors:** Amira M. Elmoslemany, Heba I. Ghamry, Abdelrahman A. Awad, Ragab I. EL-Kholy, Ibtesam S. M. Almami, Najiah M. Alyamani, Amina M. G. Zedan

**Affiliations:** 1 Nutrition and Food Science Department, Faculty of Home Economic, Al-Azhar University, Tanta, 31732 Egypt; 2 Department of Home Economics, College of Home Economics, King Khalid University, P.O. Box 960, Abha, 61421 Saudi Arabia; 3 Agricultural-Botany (Genetics) Department, Faculty of Agriculture, Al-Azhar University, Cairo, 11884 Egypt; 4 Department of Biology, College of Science, Qassim University, Buraidah, Al-Qassim Saudi Arabia; 5 Department of Biology, College of Science, University of Jeddah, Jeddah, 21493 Saudi Arabia; 6 Biological and Environmental Sciences Department, Faculty of Home Economic, Al-Azhar University, Tanta, 31732 Egypt

**Keywords:** Cell biology, Molecular biology

## Abstract

Physical and chemical changes in the natural of water may affect biological organisms. In this study, we highlight the effect of magnetized-water and microwave-water on rats’ liver tissues. Three groups of albino rats were separated. The first, rats were administered tap-water. The second, rats were administered magnetized-water. The third, rats were administered microwave-water. After two months, the results revealed a significant increase in liver functioning enzymes’ levels and bilirubin in rats administered microwave-water, compared to tap- and magnetic-water. In relation to oxidative stress, there was a significant increase and decrease in oxidative and antioxidant parameters respectively in liver tissues of rat's administrated microwave-water. At the molecular level, there was a significant down-regulation *in Metallothionein*, *CYP* genes in magnetic-water compared to tap-water. Rats administered microwave-water have shown a significant down-regulation in *GST*, *Metallothionein* and *CYP* genes’ expression, however, *Amylase* and *HDAC3* genes were significantly up-regulated, compared to the other groups. The intake of microwave-water resulted in notable histopathological changes in liver tissues. Rats administered magnetic-water showed no clear changes in their liver tissues. In summary, microwave-water induced stress and epigenetic effects compared with magnetic-water and tap-water. Also, magnetic-water produced from the higher magnetic power had no side effect on liver tissues.

## Introduction

Water is the most abundant substance on planet Earth. It is the primary component of most living organisms. Approximately, 65% of human body weight consists of water. In all practical respects, water is the only solvent in human body^1^. Due to its crucial vital rule to most living organisms, numerous physical and chemical treatments have been developed to enhance drinking water quality^2^. Magnetic-water is created by passing tap-water through magnetic field (magnetic tubes). As a consequence, water properties become extremely fertile and active; resulting in an increasement of oxygen ratio, velocity of salts and amino acid dissolution^3^. Unlike dead water, the pH of magnetic-water changes from acidity to alkalinity. Also, magnetic-water’s weight and salinity increase^4^. Magnetic-water improves the circulation of blood and oxygen, transportation of nutrients in blood, activation of enzymes, elimination of internal toxins resulted from metabolism, absorption of internal toxins and improves the blood picture and sexual hormones^3^.

It has been reported that microwave ovens usage has led to the formation of cancer-causing substances due to; (a) releasement of toxic chemicals from food packaging into food; (b) the destruction of nutrients; (c) decreased bioavailability of vitamins (such as vitamin B complex, C and E), essential minerals and lipotropic factors in food; (d) loss of natural food antioxidants. Symptoms of direct exposure to microwave ovens radiation include nervous disorders such as dizziness, headache, irritability, sleeplessness, nervous tension, anxiety, and inability to concentrate^5^. Millions of people worldwide rely on microwave ovens on daily basis for food cooking and drinks preparation.

The most important polar molecule in foods is water. Water molecules change direction with the effect of microwaves^6^. The FDA advises consumers to avoid the risk induced by boiling water in the microwave oven.^7^

This study aims to evaluate the effect of water treated with physical activity, such as magnetic and microwave apparatuses, on rats’ liver tissues at the molecular, epigenetic, histological levels and oxidative stress. The novelty of this manuscript lies in factoring magnetic and microwave treatments on the level of stress and epigenetic effects on liver tissues of rats.

## Results and discussion

Recent evidence confirmed that magnetic-water has an advantageous impact on growth parameter. Tissues of the human body are altered by exposure to non-ionized electromagnetic radiation, such as that from microwave ovens^8^. According to the current study, liver enzymes levels (AST, ALT, ALP, GGT and total bilirubin) did not significantly change in rats administered tap-water as well as rats administered magnetic-water (Table 1). However, a significant increase has been noticed in liver enzymes from rats administered microwave-water, compared to rats administered tap-water and rats administered magnetic-water. Such findings support by Al-Hilali^9^, who concluded that there were no discernible variations in AST and ALT activity of Japanese quails administered magnetically treated water in comparison with quails administered tap-water. Liver enzymes AST and ALP were not affected in mice consumed water treated with 1000 nor 2000 G^10^. According to Mahmoud^11^, water exposed to a magnetic field develops a finer and more homogeneous structure. The performance of humans, animals, and plants is significantly impacted by improved biological activity of solutions through increasing fluidity, dissolving ability of diverse elements such as minerals and vitamins. In the current study, there is no significant differences between magnetic- and tap-water. These results disagree with some studies that indicated the positive effects of magnetic water, compared to tap-water. The authors of these studies justified the positive effect with increased solubility, conductivity and mineral content of magnetic-water. Alhazmi et al.^12^ showed that animals treated with magnetized water saw weight gain, increased milk output and improved wellbeing. Additionally, Attia^13^ discovered that magnetic treatment of drinking water raised serum albumin levels (without changing globulin or albumin), globulin ratio, antioxidant status, water quality, semen quality, blood picture, liver, and kidney functions. Consequently, improved buck fertility. Regarding water treated with microwave, our results may agree with Zhang^14^. They suggested that the non-ionizing electromagnetic radiation effect (microwave oven) is linked to an increased risk of cancer, particularly in residential exposed populations within the United States. Additionally, genetic material in body tissues of those exposed to non-ionizing electromagnetic radiation from sources such as microwave ovens will change over time^8^. According to reports, microwave oven usage can result in the formation of substances that cause cancer, release of toxic chemicals from food packaging and destruction of nutrients^5^. Vitamins B complex, C, and E reduced bioavailability, as well as essential minerals and lipotropic factors in food and loss of antioxidants^5^. Microwave water treatment deleteriously impacted Japanese quails’ development and numerous physiological traits^5^. In our investigation, protein fractions' findings revealed little variations in plasma concentrations of total protein, albumin, and globulin in rats administered tap- magnetic- and microwave-water, separately.

**Table 1:** Effect of water physical treatment on liver damage parameters in the rats (Mean ± SE).

Parameters	Tap-water	Magnetic-water	Microwave-water
AST(IU/L)	130 ± 2.96^b^	136 ± 2.02^b^	198 ± 6.00^a^
ALT(IU/L)	41.33 ± 1.85^b^	40.66 ± 2.33^b^	92.66 ± 1.45^a^
ALP(IU/L)	92 ± 5.37^b^	103 ± 3.51^b^	151 ± 3.45^a^
GGT(IU/L)	11.69 ± 0.57	12.42 ± 0.68	12.85 ± 0.89
Total bilirubin (mg/dl)	0.13 ± 0.008^b^	0.14 ± 0.008^b^	0.22 ± 0.014^a^
Direct bilirubin (mg/dl)	0.05 ± 0.005^b^	0.04 ± 0.005^b^	0.11 ± 0.005^a^
Indirect bilirubin (mg/dl)	0.08 ± 0.008	0.10 ± 0.013	0.11 ± 0.012
Total protein (g/dl)	7.24 ± 0.39	8.60 ± 0.74	8.55 ± 0.49
Albumin (g/dl)	3.80 ± 0.33	5.12 ± 0.71	4.99 ± 0.11
Globulin (g/dl)	3.44 ± 0.11	3.48 ± 0.08	3.56 ± 0.39
Albumin/Globulin	1.10 ± 0.08	1.47 ± 0.20	1.42 ± 0.12

Values are presented as mean ± SE. Data with dissimilar letters [^a^ (the highest) and ^b^ (the lowest)] in the same row are significantly different at *p* ≤ 0.05. All groups were compared to each other.

**Table 2:** Effect of magnetic- and microwave-water on superoxide dismutase, catalase and oxidative parameters in liver tissues of rats (mean ± SE).

Parameters	Tap-water	Magnetic-water	Microwave-water
MDA (nmol/mg)	0.60 ± 0.06^b^	0.89 ± 0.05^b^	2.04 ± 0.45^a^
NO (umol/ L)	15 ± 1.73^b^	22 ± 1.15^b^	75 ± 9.24^a^
SOD (U/mg)	192.00 ± 15.37 ^a^	190.66 ± 2.33^a^	108.03 ± 8.33^b^
CAT (ng/mg)	10.86 ± 0.93^a^	10.89 ± 0.82^a^	3.58 ± 0.69^b^

SOD (superoxide dismutase); CAT (catalase); MDA (malondialdehyde); and NO (nitric oxide). Values with different letters (^a^ and ^b^) in each column denote significant differences.

Our results in Table (2) revealed that micro2wave-water significantly increased the level of oxidative stress (MDA and NO) and significantly decreased antioxidant enzymes’ levels (SOD and CAT) in liver tissues. In contrast, results showed non-significant variations in oxidative stress and antioxidant enzymes’ levels in liver tissues among rats administrated magnetic-water, compared to rats administered tap-water. Results from rats administered magnetic-water results disagree with Attia et al.^13^. The authors found that magnetized water significantly increased antioxidant enzymes and decreased lipid peroxidation biomarkers in rabbit bucks. This might be due to applying 4000 G, whereas in our study we applied higher magnetic power (14500 G) for water treatment. This led us to conclude that utilizing high magnetic power reduced the positive effect (without causing any visible, not detectable, deleterious effects). When rabbit bucks were administered water treated with 3600 G for 90 days, blood markers of oxidative stress (Malondialdehyde, TBARS, and lysozyme content) decreased, while total antioxidant capacity and antibody titer increased^15^. According to several studies, magnetized water can have a significant impact on the oxidant-antioxidant balance such as reducing Malondialdehyde and nitric-oxide levels, boosting superoxide dismutase enzyme activity in the heart, kidney and liver; all of which reduce the oxidative stress^16^.

In order to confirm the effect of magnetic- and microwave-water at the molecular level, expression profiles from *GST*, *Metallothionein*, *CYP*, *Amylase* and *HDAC3* genes were investigated. Authors of several articles investigated the increase, and decrease, in genes expression in response to exposure to magnetic field, not magnetic water. In *Shewanella oneidensis*, a magnetic field of 14,100 G accelerated the transcription of 21 genes, while slowing the rate of transcription in 44 other genes^17^. The accelerated genes include unknown-function genes (seven genes) and known-function genes. Two genes related to the transport and binding proteins, Methyl-accepting chemotaxis protein for cellular process function, UmuD protein for DNA metabolism, and OmpA family protein for cell envelope, were accelerated. The regulatory function includes transcriptional regulator, LuxR, LysR, and MarR family. For transcription function, there are two genes; *pnp* and Polyribonucleotide nucleotidyltransferase. In addition, *deoC* and Deoxyribose-phosphate aldolase were accelerated. There are two genes related to amino acid biosynthesis; Homoserine kinase (*thrB*) and, Diaminopimelate decarboxylase*(lysA)*. Fatty acid and phospholipid metabolism function include Acyl-CoA dehydrogenas family; Other genes include Iron-containing alcohol dehydrogenase were accelerated. On the other hand, downregulated genes include five genes related to energy metabolism (gpsA¸ petA, omcB, ccoQ, ansA) and all genes related to Amino acid biosynthesis (rpsE, rpmD, infC, rluA-2 and rpsL). Bacterioferritin subunit 1 gene (bfr1) related to transport and binding proteins function downregulated. Also, for the cellular process; fic and mine, and DNA metabolism function; Type I restriction-modification system and M subunit downregulated. Three cell envelope genes include chain length determinant protein, Polysaccharide biosynthesis protein and pile dowenregulated. For regulatory function; DNA-binding response regulator and Transcriptional regulator LysR family downregulated. In addition, there was an ATP-dependent RNA helicase and DEAD box family for downregulated transcription genes. For Purines, pyrimidines, nucleosides, and nucleotides there were carB and hpt-2 genes. Amino acid biosynthesis includes asnB gene. Peptidase, putative, and Heme exporter protein CcmD are genes related to protein fate functions that are downregulated. Other genes downregulated include Glyoxalase family protein, OmpA-like transmembrane domain protein, TPR domain protein and ISSod3, transposase.

**Figure 1:**
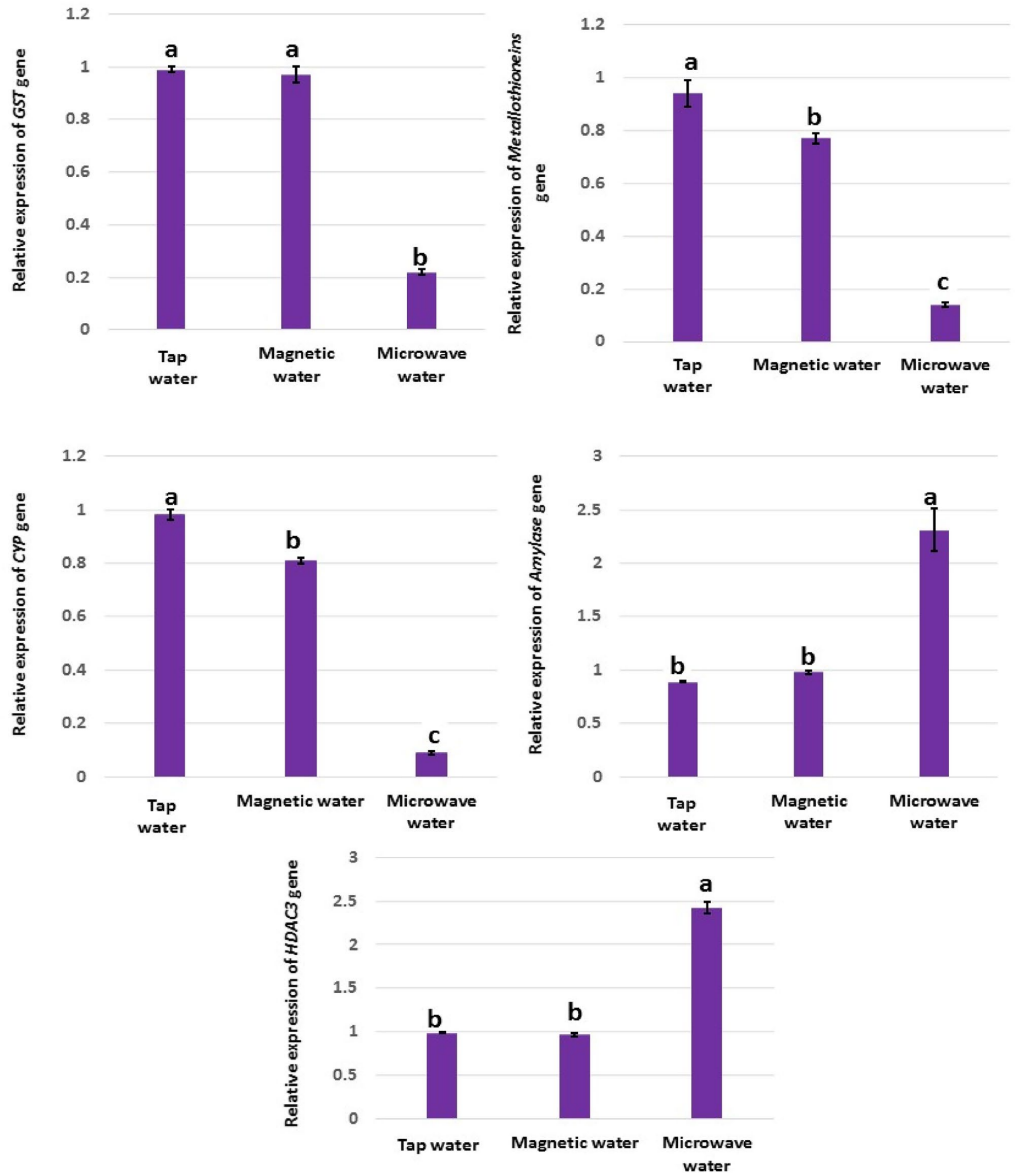
RT-qPCR expression profiles of *GST*, *Metallothionein*, *CYP*, *Amylase*, and *HDAC3* genes in liver tissues of rats administered tap-, magnetic- and microwave-water. Data were normalized to the housekeeping gene (*GAPDH*) and are expressed as mean fold-change. Tight black error bars represent standard deviation of the technical replicates. Groups with different letters denote significant differences at *p* < 0.05.

Weak magnetic fields, ranging from 0.8 to 8.0 G, promoted the transcription of the *c-myc* gene in mice and humans^18^. It was reported that magnetic field interferes with normal genome function at the level of gene expression (magnetic field-induced expression)^19^. In our study, magnetic-water significantly reduced gene expression of *Metallothionein* “Fig. 1”, and this disagrees with Chater et al.^20^. The authors found that female rats' plasma *Metallothionein* activities were boosted in response to short-term exposure to a magnetic field; and suggested that such increases in *Metallothionein* activities indicating that biosynthesis of *Metallothionein* might have been induced by stress^20^. The fact that magnetic-water, in our results, did not induce the stress that was induced in presence of direct magnetic-field agrees with Chater et al*.*^20^. Previous findings indicated that magnetic-water had no negative effect on liver’s health. We can clearly see that the indirect exposure to magnetic-field (magnetic-water) was not harmful. Also, down-regulation of *CYT* gene disagrees with the increased levels of *CYT* in the study reported by Patruno^21^ in response to magnetic-field, not magnetic-water in our study as discussed earlier. This is the first time to study magnetic-water and its effect on the expression of genes investigated. In contrast, there was no significant expression differences, between magnetic-water and tap-water, in the remainder of the investigated genes (*GST, Amylase* and *HDAC3*). When biological material is exposed to magnetic radiation, it absorbs a certain amount of energy, which depends on the dielectric properties of the biological material^22^. This non-ionizing electromagnetic radiation is absorbed, at the molecular level, and shows up as changes in the molecules’ vibrational energy or heat. Microwaves cause different biological effects; through adding ions in different locations. As a consequence, the speed and/or direction of biochemical reactions is modified^23^. Cell treatment with and without water is different as described by Szopińska and Dorna^24^. In addition, the different results show that water "the different factor in two experiment" had an obvious effect on the living cell. Significant down-regulation of *GST*, *Metallothionein* and *CYP* genes and up-regulation of *Amylase* and *HDAC3* genes induced by microwave-water, compared to tap-water, indicated the deleterious effects of microwave treatment. These effects could be due to the changes in the water properties such as pH, conductivity and mobility of water molecules^25^. We also have noticed the precipitation of salt after boiling water in the microwave oven; this precipitation due to attaining supercritical condition^26^ and it could be the main reason that causes the negative effects of microwave treatment. Along with other changes in the normality of water that were mentioned by other authors led to vital changes induced in living cells. The fact that water is highly dipolar is what makes it able to dissolve salts. This makes it possible for water molecules to form spheres around the separated salt ions. As the temperature goes up, structure of the solution (hydrogen-bonding network and the clustering of water molecules around solutes) slowly changes. This is caused by an increase in the molecules’ kinetic energy and a corresponding decrease in their molecular interaction. The effective electrostatic force, associated with ion charges, diminishes with temperature at a much slower pace than the water dipole moment does. As a result, the hydrogen-bonded network weakens and cannot provide adequate protection from the salt ions^26^. These alterations in tap-water properties could lead to epigenetic effects on the genes. This is very clear in the highly induced regulation of *HDAC3* gene by microwave-water*. HDAC3* belongs to genes that play a major role in epigenetic effects. *HDAC3* encodes for a protein that belongs to histone deacetylase. Microwave-water induced up-regulation in this gene, compared to tap- and magnetic-water. Therefore, we can conclude that microwave-water had a role in epigenetic effect.

**Figure 2:**
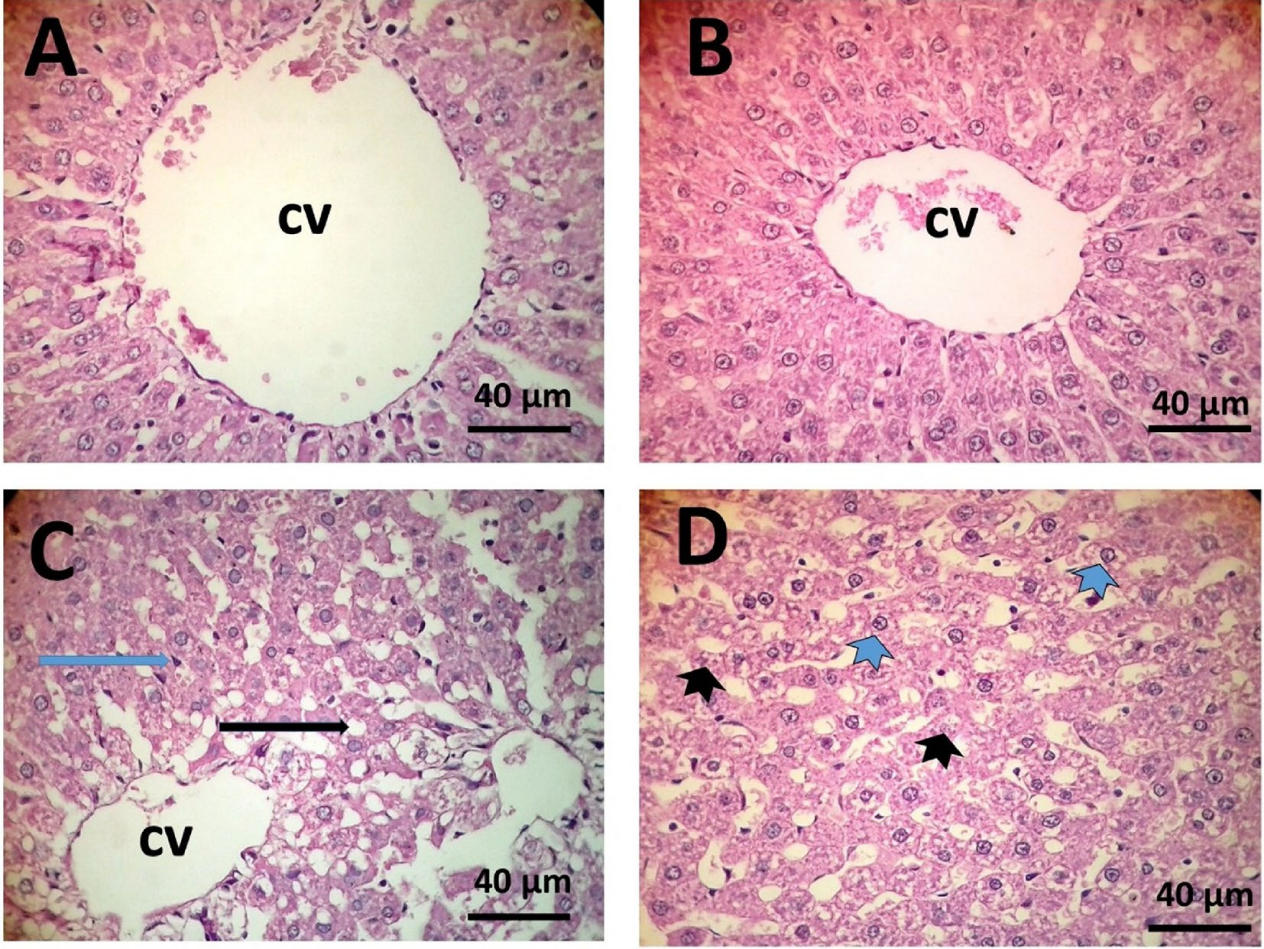
Liver tissue microscopic sections showing normal appearance of hepatic cells radiating from the central vein, dilatation in the central vein (cv) in rats administered tap-water group (**A**); normal appearance of hepatic cells and central vein in rats administered magnetic-water group (**B**); droplet of lipids (black arrow) increase in Kupffer cells (blue arrow) karyolysis of nuclei (black arrowhead), vacuolation of cytoplasm (blue arrowhead) in rats administered microwave-water group (**C** and **D**). (40×) Scale bar: 40 µm.

Histological examination of liver tissues in rats administered tap-water revealed the presence of normal hepatocytes and dilatation in the central vein (Fig. 2A). Similarly, administration of magnetic-water resulted in normal liver tissues (Fig. 2B). In contrast, liver tissues from rats administered microwave-water showed various histological changes such as droplet of lipid, increase in Kupffer cells, karyolysis of nuclei and vacuolation of cytoplasm “Fig. 2C,D”. According to the best of our knowledge, there are no publications that have investigated the side effects of microwave-treated water. The histopathological alterations induced by microwave-water could be attributed to an increase in reactive oxygen species (MDA and No) and decrease in antioxidant activities (SOD and CAT). These results disagree with Mansoor et al.^27^; they reported negative effects on liver tissues of *Cyprinus carpio* fish. Studies on the histological effects of magnetized water on animals, especially rat liver, appear to be scarce. However, Hassan et al.^28^ reported that prolonged exposure to magnetized water at 0.2 Tesla may result in liver damage of *Scortum barcoo* fish; based on histopathological observations of the liver. Aboulfotoh et al.^29^ demonstrated the impact of water (treated with either a magnetic field or microwave oven) on bone minerals and concluded that magnetized water has a beneficial impact on bone minerals. However, they also reported that microwave-treated water had an unfavorable effect on bone minerals; leading to decreased minerals than those reported in the control and magnetic water groups.

In the last, we can conclude that some physical, not all, can change the nature of water and consequently affect the organism. This study concludes that microwave-treated water changed the liver function enzymes, oxidative stress, and antioxidant parameters. In addition, it changed the gene expression for important genes. Magnetically treated water had no side effects compared with normal water.

## Methods

### Experimental design

This study was conducted at the animal house of Delta University of Science and Technology. The experimental design was approved by Delta University's ethical committee. Twenty-one *Sprague Dawley* albino male rats (150 ± 10 g; 10 to 12 weeks old) were housed in cages under standard conditions (12 h light/12 h dark cycle; 23 ± 2 °C and 60% humidity). Rats were fed on pelleted diet containing the following nutritional ingredients: soybean meal (11%), sunflower oil (15%), yellow corn (49%), concentrate mixture (10%), wheat bran (10%), common salt (0.5%), molasses (3%), dicalcium phosphate (0.1%), ground limestone (0.2%), dl-methionine (0.7%), lysine (0.2%) and mineral-vitamin premix (0.3%)^30^. Rats had free access to water and food. Upon rats’ adaptation, they were randomly distributed into three groups; each group contained seven rats. Rats of the first group (negative control) administered natural tap-water for the entire duration of the experiment (two months). The second group of rats (magnetic-water group) administered magnetic-water produced from natural drinking water by passing through a 14,500 Gauss (G) magnetic field within a device (magnetizer) that was purchased from Delta Water Company (Alexandria, Egypt). (the device connected to the tap, we let water pass for two min. and then receive the water in a two-liter flask with a slow flow for 5 min.; rats drank magnetic water ad libitum) Third group of rats (microwave-water group) administered tap-water treated with microwave oven (Sharp Microwave 34 Liters, Stainless steel, 60 Hz, Model: R-77AS) for 10 min in a glass beaker until boiling and then cooled at room temperature^29^ (the time was approximately ten minutes from put the water into the oven till boiling).

### Sampling

By the end of the experiment timeline (second month after adaptation), rats went through overnight fasting process. Rats were then euthanized by exsanguination. Blood samples were collected from the portal veins of the liver. Sera were separated by centrifugation at 4000 rpm for 10 min. The liver was immediately removed, instantly washed with isotonic saline, and weighed. Then, liver was split into three parts; (a) first part was kept in − 80 °C for further RNA and molecular analysis; (b) second part was fixed for further histopathological examination in formalin 10%; (c) third part was homogenized for further antioxidant analysis.

### Biochemical analysis

Alkaline phosphatase (ALP), aspartate transaminase (AST) and alanine transaminase (ALT) activities were measured using kits from Diamond-Diagnostics (Cairo, Egypt). Kits from BioMed-Diagnostics were utilized to measure gamma-glutamyl transpeptidase (GTP), albumin and bilirubin (EGY-CHEM, Cairo, Egypt)^31^.

### Oxidative markers and antioxidants in liver tissues

Antioxidants and oxidative markers activity measurement took place on homogenized tissues from liver. One gram of liver tissues was homogenized in 9 ml ice-cold phosphate-buffered saline, followed by centrifugation at 10,000 rpm under cooling (4 °C). Estimating various antioxidants levels took place on the supernatant via colorimetric and ELISA methodology, using Rayto ELISA microplate reader (RT-2100C, China). Lipid peroxidation via thiobarbituric acid-reactive substances (TBARS) methodology^32^. NO, Superoxide dismutase (SOD) and Catalase (CAT) were estimated according to Archer^33^, Paoletti,^34^ and Aebi^35^, respectively.

### RT-qPCR analysis of gene expression in hepatic tissue

Total RNA from samples representing each individual in each group was extracted, using Direct-zol™ RNA MiniPrep Plus kit (Catalog # R2071, ZYMO Research Corp., USA) in three technical replicates. Extracted RNA quality and concentrations were evaluated using NanoDrop™ (ND-1000) spectrophotometer (Thermo Scientific, Waltham, MA, USA). The SuperScript™ IV One-Step RT-PCR kit (Catalog Number 12594100; Thermo Scientific, Waltham, MA, USA) was used to generate cDNA from extracted RNA; followed by diluting all cDNA samples to the same concentrations using NanoDrop™ (ND-1000) spectrophotometer. Real-time qPCR reactions included cDNA as a template, QuantiTect™ SYBR Green qPCR Master Mix and gene-specific primers. Primers were designed using web-tool Primer3web™ (version 4.1;^36^) utilizing sequences published from the rat genome project (Table 3). Quantitative real-time PCR reactions were conducted using the StepOnePlus™ system (Applied Biosystems, USA). Threshold cycle (Ct) values of targeted genes were normalized to Ct values of the internal control (*GAPDH*)^37^.

**Table 3:** Real-time qPCR primers.

Gene	Forward primer	Reverse primer
*GST*	TTCAAGGCTCGCTCAAGTCCAC	CTTGATCTTGGGGCGGGCACTG
*Metallothionein*	CACAGATGGATCCTGCTCCT	AAGTGTGGAGAACCGGTCAG
*CYP1a2*	TCCACATTCCCAAGGAGTGCT	TAAGAAACCGCTCTGGGCG
*AMYLASE*	TGGCCTTCTGGATCTTGCACTC	AGGCTGACCGTTGACTACATTCCT
*HDAC3*	CGTCCGAAATGTTGC	GAAGTTCCTCACTAATGG
*GAPDH*	GGTGATGCTGGTGCTGAGTA	GGATGCAGGGATGATGTTCT

### Histopathology examinations

Small portions of the liver were fixed in 10% formalin solution and dehydrated in 70–100% ethanol gradient dilutions, then cleared in xylene and encapsulated in paraffin for further sectioning. Liver sections were stained with Eosin and Hematoxylin dyes, according to Suvarna et al*.*^38^.

### Statistical analysis

One-way analysis of variance (ANOVA) and Duncan tests were utilized to conduct statistical analysis (SPSS software, version 22.0). Statistical results were reported as the mean ± standard error (SE). Differences between means are deemed statistically significant at *p* < 0.05.

### Ethics declarations

The experiment was carried out under Egyptian ethical codes for studies on experimental animals and approved by the Animal Ethical Committee, Delta University’s ethical committee, Egypt (number FODMRC 2,021,000,100, Date 20–10–2021) and all experiments were performed by relevant guidelines and regulations. The study was carried out in compliance with the ARRIVE guidelines.

## Data Availability

All data generated or analyzed during this study are included in this published article.
